# Cystatin C and lactoferrin concentrations in biological fluids as possible prognostic factors in eye tumor development

**DOI:** 10.3402/ijch.v72i0.21087

**Published:** 2013-08-05

**Authors:** Mariya A. Dikovskaya, Alexandr N. Trunov, Valeriy V. Chernykh, Tatyana A. Korolenko

**Affiliations:** 1 Institute of Physiology, Siberian Branch of Russian Academy of Medical Sciences, Novosibirsk, Russia; 2 The S.N. Fyodorov Federal State Complex “Eye Microsurgery” (Novosibirsk Branch), Novosibirsk, Russia; 3 Research Center of Clinical and Experimental Medicine of SB Branch

**Keywords:** choroidal melanoma, cystatin C, lactoferrin, eye biological fluids, tears, serum

## Abstract

**Objectives:**

To investigate the possible role of cystatin C in eye biological fluids locally and in serum and lactoferrin revealing anti-tumor activity in eye tumor development.

**Background:**

The increased number of eye tumors was registered recently not only in the countries with high insolation, but also in the northern countries including Russia (11 cases per million of population). Search for new biological markers is important for diagnosis and prognosis in eye tumors. Cystatin C, an endogenous inhibitor of cysteine proteases, plays an important protective role in several tumors. Lactoferrin was shown to express anti-tumor and antiviral activities. It was hypothesized that cystatin C and lactoferrin could serve as possible biomarkers in the diagnosis of malignant and benign eye tumors.

**Study design:**

A total of 54 patients with choroidal melanoma and benign eye tumors were examined (part of them undergoing surgical treatment). Serum, tear fluid and intraocular fluid samples obtained from the anterior chamber of eyes in patients with choroidal melanoma were studied.

**Methods:**

Cystatin C concentration in serum and eye biological fluids was measured by commercial ELISA kits for human (BioVendor, Czechia); lactoferrin concentration – by Lactoferrin-strip D 4106 ELISA test systems (Vector-BEST, Novosibirsk Region, Russia).

**Results:**

Cystatin C concentration in serum of *healthy* persons was significantly higher as compared to tear and intraocular fluids. In patients with choroidal melanoma, increased cystatin C concentration was similar in tear fluid of both the eyes. Lactoferrin level in tear fluid of healthy persons was significantly higher than its serum level. Significantly increased lactoferrin concentration in tear fluid was noted in patients with benign and malignant eye tumors.

**Conclusion:**

Increased level of cystatin C in tear fluid seems to be a possible diagnostic factor in the eye tumors studied. However, it does not allow us to differentiate between malignant and benign eye tumors. Similar changes were noted for lactoferrin in tear fluid.

Cystatins belong to the group of endogenous inhibitors of cysteine proteases, which are important in clinical and experimental medicine (1,2). The main functions of cystatins are related to the inhibition of cysteine proteases, cell proliferation, cell migration and cell differentiation (3,4). Cystatins A and B (belonging to type 1) are mainly intracellular inhibitors; cystatins C, D, E/M, F, G, S, SN and SA are extracellular cystatins (belonging to type 2). Cystatins SA, S and SN were found primarily in saliva. Cystatins S and SN can also be expressed in tears, urine and seminal fluid (4). Cystatin C was shown to play a role in protein catabolism, cancer development, regulation of hormone processing and bone resorption, modulating inflammation (5,6). Increased cystatin C level has been found in the *serum* of patients with several inflammatory diseases, such as colorectal tumors with metastases (7). Cystatin C as well as cystatins B, SN, SA and S was identified in tear fluid (4). Among the cystatin type 2 superfamily, cystatin C is the most studied; it is expressed in brain, thymus and epididymis. Cystatin C is produced at a constant rate by all types of cells and localized mainly in extracellular fluids; the high concentration was shown in cerebrospinal fluid, and in lower concentrations in milk, synovial fluid, serum, urine and bile (8,9). In eye fluids (less studied as compared to other biological fluids), cystatin C as a secreted protein was suggested to play the role of inhibiting all cysteine proteases, such as cathepsins B, L, S (10–12). It was shown recently that some members of the cystatin superfamily (cystatin A) have antiapoptotic properties linked with neoplastic changes in squamous cell epithelium. Therefore, it has been proposed as a diagnostic and prognostic marker of lung cancer (13). Recently, cystatin C was suggested as a prognostic factor in multiple myeloma (14), in the diagnostics of early-stage and inflammatory breast cancer (15). However, the role of cystatin C in eye tumors has not been studied in detail.

Lactoferrin is a 78,000-Da metal-binding single-chain glycoprotein found in milk, tear fluid and other biological exocrine secretions (16,17). Lactoferrin has bacteriostatic properties in vitro and some anti-tumor activity (16,18). Since the human lactoferrin gene has been cloned, overexpression and large-scale lactoferrin production are now possible.

Choroidal melanoma is one of the most aggressive malignant and most observed among eye tumors. Until now, there have been difficulties in differential diagnostics of choroidal melanoma with numerous eye tumors, mainly with benign tumors, as well as with so-called pseudo-tumorous disorders. Pseudo-tumors included disciform macular degeneration, sub-retinal hemorrhage, Coats’ disease (very rare congenital, nonhereditary eye disorder) and so on. Therefore, the search for possible new tumor biomarkers among different types of eye tumors is still important. *The aim* is to investigate the role of cystatin C in eye tear fluid and anterior camera of eye *locally*, and in serum and lactoferrin, revealing anti-tumor activity in eye tumor development (choroidal melanoma).

## Materials and methods

### Design of the study

A total of 43 patients with choroidal melanoma and 11 with benign eye tumors and pseudo-tumorous disorders (undergoing examination and treatment in Fedorov's Centre for Eye Surgery) and 24 controls (healthy persons, medical doctors) were enrolled in this study. All patients were undergoing standard ophthalmologic examinations, including visiometric analysis, perimetry and visual field research, tonometry, biomicroscopic examination, ophthalmoscope examination and ultrasound exam (B-scanning). In several special cases, the following additional methods were used: ultrasound eye biomicroscopy, optical coherent tomography of retina, duplex eye scan with the dopplerography examination of vessels.

The group of patients with *choroidal melanoma* included 43 persons aged 28–80 (56.9±15 years; males: 15, females: 28). Their vision was from 0 to 1.0. In all patients, ultrasound examination revealed plus-tissue (with low or middle echo-density) growing from the vessels. In most of the patients (42), the tumor process was noted in one eye, in 1 patient – in both eyes (simultaneously with breast cancer). The eye tumor length (height) was from 1.2 to 14.7 mm, diameter of the tumor basement – from 8.2 to 27.6 mm. In 17 cases with large-sized tumors, surgery was carried out (enucleating). Histological study revealed the appearance of round-cellular melanoblastoma (7 cases), spindle-cell malignant melanoma (6 cases), epithelial type of melanoblastoma (2 cases) and melanoblastoma of the mixed type (2 cases). *Benign eye tumors* and *pseudo-tumors* group included 11 persons, aged 42–74 (62.3±9.8 years); among them 1 male and 10 females. The patients with the following diagnosis were included in this group: age-related macular degeneration – 3 (vision from 0.001 to 0.1), sub-retinal hemorrhage – 3 (vision from 0.001 to 0.01), iris-*nevus* syndrome (nevus of iridescent) – 2 (vision 1.0) and nevus chorioidea – 3 patients (vision 1.0). Ultrasound scanning revealed plus-tissue syndrome, with increased tissue from 0.5 to 4.7 mm with different echo-densities. In 4 patients, duplex scanning with dopplerography examination did not reveal any plus-tissue and changes in blood vessels. *Control group* – 24 healthy persons without ophthalmologic disorders, 20–49 years old (22±15) were used as a control. Four patients with cataracts aged 58–81 were included in this group when uptake of anterior camera fluid was carried out during surgery. In addition, as a control for the study of serum cystatin C, 10 healthy persons aged 51–65 years were included.

### Sample collection

Tear samples were collected using 10-µL capillary tubes without touching the eye globe or the lid. Intraocular fluid was taken from anterior eye camera during surgery. Serum was obtained after centrifuging blood samples at 3000*g* for 20 min at 4°C (Eppendorf centrifuge 5415R, Hamburg, Germany) and stored at −70°C until analysis.

Cystatin C concentration in eye biological fluids and serum was measured by commercial ELISA kits for human cystatin C (BioVendor, Czechia). The antibodies used in ELISA are specific for human cystatin C. Analytical limit of detection (ALD) of cystatin C was 0.2 ng/mL. Detection of cystatin C did not interfere with hemoglobin (1.0 mg/mL), bilirubin and triglycerides (5.0 mmol/L). The results were expressed in nanogram per milliliter.

Lactoferrin concentration was assayed by commercial ELISA kits (Vector-BEST, Novosibirsk Region, Russia) according to the producer's recommendations. The results were expressed in nanogram per milliliter.

### Statistical analysis

All values were reported as the mean±SEM. The results were analyzed for statistically significant differences, using Mann–Whitney criteria and Student's t-test.

## Results

Cystatin C concentration in the serum of *healthy* persons (950.4±21.2 ng/mL) was significantly higher (p<0.001) as compared to tear (371.3±25.7 ng/mL) and intraocular fluids (765.6±91.1 ng/mL, p<0.05). Among eye biological fluids studied, cystatin C level was higher in intraocular anterior camera fluid as compared to tears (p<0.001) (Table I). Therefore, concentration of cysteine protease inhibitor was presented as follows: serum > intraocular fluid > tears.

**Table I:** Cystatin C concentrations (ng/mL) in biological fluids in choroidal melanoma and benign eye tumors (m±SEM)

Biological sample	Control	Choroidal melanoma	Benign tumors and pseudo-tumorous disorders
Tear fluid (eye with tumor)	–	461.3±17.2** # (n=42)	471.5±15.5** (n=11)
Tear fluid (eye without tumor)	371.3±25.7# (n=24)	496.6±23.9** # (n=33)	477.5±17.4** (n=11)
Anterior camera fluid	765.6±91.1# (n=4)	585.9±72.1* # (n=11)	–
Blood serum	950.4±21.2 (n=10)	1212.9±92.5* (n=16)	778.8

* p<0.05

** p<0.001 versus the appropriate control.

# p<0.001 versus serum of the same group. The number of patients is given in parenthesis.

### Choroidal melanoma

Tear fluid cystatin C level in both the eyes increased (p<0.001), whereas concentration of this inhibitor in anterior camera fluid decreased (p<0.05) as compared to the control group (Table I). In choroidal melanoma, cystatin C level was increased similarly in the tear fluid of both the eyes (with and without tumor) versus control (Table I). Cystatin C concentration is *serum* was significantly increased (p<0.05) in choroidal melanoma. Therefore, in patients with choroidal melanoma cystatin C concentration was elevated in the tear fluid of both the eyes (with tumor and without tumor), whereas in serum similar changes were less prominent.

Lactoferrin concentration in tear fluid of healthy persons (Table II) was significantly, approximately 10 times, higher than lactoferrin levels in the serum of the control (800–1200 ng/mL) group. In anterior camera fluid of the control group, lactoferrin concentration was significantly (p<0.05) lower compared to that of the tear fluid and serum (Table II). Increased lactoferrin concentration was noted in tear fluid in patients with both benign (p<0.05) and malignant eye tumors (p<0.05) and in anterior camera fluid (p<0.05) in choroidal melanoma (Table II). In patients with choroidal melanoma, an increased level of lactoferrin was shown both in tear fluid of a tumor-damaged eye and tear fluid of an eye without tumor (Table II). There was no difference between increased level of lactoferrin in tear fluids of patients with choroidal melanoma and benign eye tumors (Table II). The role of lactoferrin in eye tumor has not been studied enough until now. Recently, in some other tumors (breast cancer phenotypes), lactoferrin was shown to contribute to the development and invasiveness of this disorder (19).

**Table II:** Lactoferrin concentrations (ng/mL) in biological fluids in choroidal melanoma and benign eye tumors (m±SEM)

Biological sample	Control	Choroidal melanoma	Benign tumors and pseudo-tumorous disorders
Tear fluid (eye with tumor)	–	13,388±274* (n=22)	13,556±338* (n=9)
Tear fluid (eye without tumor)	11,886±213 (n=20)	13,564±301* (n=22)	13,751±359* (n=9)
Anterior camera fluid	342±27 (n=6)	581±53* (n=6)	–

* p<0.05 versus control. The number of patients is given in parenthesis.

## Discussion

The search for new non-invasive markers and predictors of tumors of different localization is important for clinical oncology, especially in rare tumors, such as melanoblastoma chorioidea, where it is impossible to get biopsy material. Moreover, this tumor can be treated only surgically (enucleation) at present, and early diagnosis is essential. Until now, the biological markers of melanoblastoma chorioidea are not known. The tear fluids have a special interest (availability of this biological fluid) for searching biological markers. According to hypothesis, many tumors to secrete both proteases of different classes and their endogenous inhibitors; disturbances in the balance of proteases/inhibitors play the important role in tumor growth and metastasizing processes (1). Therefore, the search among secreted proteins of biological fluids is of special interest. The changes in balance protease/inhibitors (increased expression and activity of proteases and decreased level of inhibitors) were shown in several malignancies as a result of increased consumption of inhibitors (7). Cystatin C was shown to be implicated in the invasiveness of human glioblastoma cells and as a result, sense transcripts of cystatin C may prove useful in cancer therapy. However, in some hemoblastoses (lymphoma, lymphogranulomatosis) opposite data were obtained: *increased* serum cystatin C level was revealed, and – *decreased* cystatin C concentration in acute leukosis (9). Cystatin C belongs to known secreted inhibitors among the cystatin superfamily, which includes both extracellular and intracellular types of inhibitors of cysteine proteases. Cystatin C was shown to be secreted by different cell types, especially by activated macrophages, as well as by some tumor cells (3).

The tear proteins play an important role in maintaining the ocular surface, and changes in their components may reflect the disturbances in the health of the ocular surface. Tears can be used to study the proteomic responses in patients with inflammatory eye disorders, such as fungal keratitis (20) and possibly in tumor eye disorders. The proteomic analysis of tear fluids in healthy persons already showed some promising results in eye research; 491 proteins were identified, revealing a large number of proteases and protease inhibitors, such as cystatins B, C, SA, SN, S (21). In our study, cystatin C and lactoferrin levels in tear fluid of patients with choroidal melanoma did not relate with the size of the tumor and their localization. Further studies are necessary to reveal the role of other types of cystatins (such as cystatins CN, S) in eye fluids, which can be useful in differential diagnosis of eye malignancies. Cystatin C is present in high concentrations in the normal human and rat retinas and similarly expressed in normal human and mouse retinas; it was suggested that cystatin C could be involved in the regulation of photoreceptor degradation in retinas (11). Cystatin C also reveals some immunomodulatory activity, playing a role in antigen presentation (4,22). According to some experimental data, uptake of cystatin C by eye cells has been shown (11). In experiments in mice and rats, it was shown that cystatin C administered intravitreally in vivo is taken up into cells of the corneal endothelium and epithelium, the epithelial cells lining the ciliary processes and into cells in the neuroretina and the retinal pigment epithelium (11,12). The active, temperature-dependent uptake of cystatin C into several cell types (the same that contain endogenous cystatin C) in the cornea, ciliary body and retina was noted (11). The uptake of cystatin C indicates that the inhibitor may exert biological functions in intracellular compartments. It is also possible that this uptake system may regulate the extracellular levels of cystatin C in the eye (11,12). Possibly, there is the potential to use cystatins in the therapy of different eye disorder (including eye tumor) to increase the host cell resistance.

## Conclusion

Tear cystatin C and lactoferrin level, increased in malignant and benign eye tumors, seems to be a perspective for diagnostics in these disorders. However, it is impossible to differentiate choroidal melanoma and benign eye tumors according to the level of cystatin C and lactoferrin in eye tear fluids.

## References

[1] Mussap M, Plebani M (2004). Biochemistry and clinical role of human cystatin C. Crit Rev Clin Lab Sci.

[2] Magister S, Obermajer N, Mirkovic B, Svajger U, Renko M, Softic A (2012). Regulation of cathepsins S and L by cystatin F during maturation of dendritic cells. Eur J Cell Biol.

[3] Kos J, Lah TT (1998). Cysteine proteinases and their endogenous inhibitors: target proteins for prognosis, diagnosis and therapy in cancer (review). Oncol Rep.

[4] Keppler D (2006). Towards novel anti-cancer strategies based on cystatin function. Cancer Lett.

[5] Turk V, Bode W (1991). The cystatins: protein inhibitors of cysteine proteinases. FEBS Lett.

[6] Bobek LA, Levine MJ (1992). Cystatins – inhibitors of cysteine proteinases. Crit Rev Oral Biol Med.

[7] Kos J, Krasovec M, Cimerman N, Nielsen H, Christensen IJ, Brunner N (2000). Cysteine protease inhibitor stefin A, stefin B, and cystatin C in sera from patients with colorectal cancer; relation to prognosis. Clin Cancer Res.

[8] Mulaomerovic A, Halilbasic A, Cickusic E, Zavasnik-Bergant T, Begic L, Kos J (2007). Cystatin C as a potential marker for relapse in patients with non-Hodgkin B-cell lymphoma. Cancer Lett.

[9] Korolenko TA, Cherkanova MS, Gashenko EA, Johnston TP, Bravve IY, Cohen J, Ryseck LP (2011). Cystatin C, atherosclerosis and lipid-lowering therapy by statins. Cystatins, protease inhibitors.

[10] Barka T, Asbell PA, van der Noen H, Prasad A (1991). Cystatins in human tear fluid. Curr Eye Res.

[11] Wasselius J, Håkansson K, Johansson K, Abrahamson M, Ehinger B (2001). Identification and localization of retinal cystatin C. Invest Ophthalmol Vis Sci.

[12] Wassélius J, Johansson K, Hakansson K, Abrahamson M, Ehinger B (2005). Cystatin C uptake in the eye. Graefes Arch Clin Exp Ophthalmol.

[13] Butler MW, Fukui T, Salit J, Shaykhiev R, Mezey JG, Hackett NR (2011). Modulation of cystatin A expression in human airway epithelium related to genotype, smoking, COPD and lung cancer. Cancer Res.

[14] Terpos E, Katodritou E, Tsiftsakis E, Kastritis E, Christoulas D, Pouli A (2009). Cystatin C is an independent prognostic factor for survival in multiple myeloma and is reduced by bortezomib administrayion. Haematologica.

[15] Decock J, Obermajer N, Vozelj S, Hendrickx W, Paridaens R, Kos J (2008). Cathepsin H, cathepsin X and cystatin C in sera of patients with early-stage and inflammatory breast cancer. Int J Biol Markers.

[16] Tomita M, Takase M, Bellamy W, Shimamura S (1994). A review: the active peptide of lactoferrin. Acta Paediatr Jpn.

[17] Mann DM, Romm E, Migliorini M (1994). Delineation of the glycosaminoglycan-binding site in the human inflammatory response protein lactoferrin. J Biol Chem.

[18] Wei M, Xu Y, Zou Q, Tu L, Tang C, Xu T (2012). Hepatocellular carcinoma targeting effect of PEGylated liposomes modified with lactoferrin. Eur J Pharm Sci.

[19] Ha NH, Nair VS, Reddy DN, Mudvari P, Ohshiro K, Ghanta KS (2011). Lactoferrin-endothelin-1 axis contributes to the development and invasiveness of triple-negative breast cancer phenotypes. Cancer Res.

[20] Ananthi S, Chitra RT, Bini R, Prajna NV, Lalitha P, Venkataswamy G (2008). Comparative analysis of the tear protein profile in mycotic keratitis patients. Mol Vis.

[21] de Souza GA, Godoy MF, Mann M (2006). Identification of 491 proteins in the tear fluid proteome reveals a large number of proteases and protease inhibitors. Genome Biol.

[22] Mirkovic B, Premzl A, Hodnik V, Doljak B, Jevnikar Z, Anderluh G (2009). Regulation of cathepsin B activity by 2A2 monoclonal antibody. FEBS J.

